# External validation and comparison of clinical prediction models for cisplatin-associated acute kidney injury: a single-centre retrospective study

**DOI:** 10.1186/s40780-025-00471-0

**Published:** 2025-09-29

**Authors:** Kazuki Saito, Satoru Nihei, Junichi Asaka, Kenzo Kudo

**Affiliations:** 1 Department of Pharmacy, Iwate Medical University Hospital, 2-1-1, Idaidori, Yahaba-Cho , Shiwa-Gun, Iwate 028-3695 Japan; 2 Division of Clinical Pharmaceutics and Pharmacy Practice, Department of Clinical Pharmacy, School of Pharmacy, Iwate Medical University, 1-1-1, Idaidori, Yahaba-Cho, Shiwa-Gun, Iwate 028-3609 Japan

**Keywords:** Clinical decision rules, Cisplatin, Acute kidney injury, Validation study

## Abstract

**Background:**

Cisplatin-associated acute kidney injury (C-AKI) is a major complication of cisplatin therapy. Although two clinical prediction models have been developed for the US population, their external validity in the Japanese population remains unclear. This study aimed to evaluate the external validity of these models and compare their predictive performances in a Japanese cohort.

**Methods:**

We assessed the performance of two C-AKI prediction models developed by Motwani et al. and Gupta et al. in a retrospective cohort of 1,684 patients treated with cisplatin at Iwate Medical University Hospital. C-AKI was defined as a ≥ 0.3 mg/dL increase in serum creatinine or a ≥ 1.5-fold rise from baseline. Severe C-AKI was defined as a ≥ 2.0-fold increase or renal replacement therapy initiation. Model performance was evaluated using discrimination (area under the receiver operating characteristic curve [AUROC]), calibration, and decision curve analysis (DCA). Logistic recalibration was applied to adapt the model to the local population.

**Results:**

The discriminatory performance for C-AKI was similar between the Gupta and Motwani models (AUROC, 0.616 vs. 0.613; *p* = 0.84). However, the Gupta model showed better discrimination of severe C-AKI (AUROC, 0.674 vs. 0.594; *p* = 0.02). Both models exhibited poor initial calibrations, which improved after recalibration. The recalibrated models yielded a greater net benefit in the DCA, with the Gupta model demonstrating the highest clinical utility in severe C-AKI.

**Conclusions:**

Both models demonstrated discriminatory ability, with the Gupta model showing particular utility in predicting severe C-AKI. Given the observed miscalibration, recalibration is essential before applying these models in Japanese clinical practice.

**Supplementary Information:**

The online version contains supplementary material available at 10.1186/s40780-025-00471-0.

## Background

Cisplatin (CDDP) is an important chemotherapeutic agent with proven efficacy against various solid tumours [1]. One of its major dose-limiting toxicities is nephrotoxicity [2], with cisplatin-associated acute kidney injury (C-AKI) occurring in 20%–30% of patients and in approximately 10% even after the first dose [3, 4]. C-AKI is associated with poor renal outcomes [4], treatment interruptions, poor prognosis [5–7], prolonged hospital stays, and increased healthcare costs [8, 9]. Preventive strategies, such as hydration, forced diuresis with mannitol or furosemide [10], and magnesium supplementation [11], are commonly employed to mitigate CDDP-induced nephrotoxicity. However, these measures did not completely eliminate the risk of C-AKI.

Given the frequency and potential severity of C-AKI, assessing the associated risks in advance is important. Clinical prediction models, tools that estimate the probability of a specific outcome using multiple predictors, are widely used in medical practice. In 2018, Motwani et al. developed a prediction model for C-AKI using data from the US population [12]. This scoring system estimates C-AKI risk based on readily available clinical variables, such as age, CDDP dose, serum albumin level, and history of hypertension. In 2024, Gupta et al. proposed a new model that incorporated additional predictors, including blood cell counts, haemoglobin levels, and serum magnesium concentration (Table 1) [5]. The Motwani model included mild AKI (≥ 0.3 mg/dL increase in serum creatinine), whereas the Gupta model targeted severe AKI (≥ 2.0-fold increase or renal replacement therapy (RRT) initiation), which is clinically more critical. The Gupta model demonstrated superior discriminatory performance compared to the earlier model developed by Motwani et al. in severe C-AKI.

**Table 1:** Clinical prediction models for cisplatin related acute kidney injury

	Motwani et al	Gupta et al
Definition of AKI	Creatinine ≥ 0.3 mg/dL in 14 days	Creatinine ≥ 2.0-fold or RRT in 14 days
Predictors		
Age (years)	≤ 60: 0 points	≤ 45: 0 points
	61–70: 1.5 points	46–60: 2.5 points
	> 70: 2.5 points	61–70: 3.5 points
		> 70: 4.5 points
Hypertension	2 points	1 point
Diabetes	–	1 point
Smoker	–	1 point
CDDP dosage (mg)	≤ 100: 0 points	≤ 50: 0 points
	101–150: 1 point	51–75: 2 points
	> 150: 3 points	76–100: 2.5 points
		101–125: 3 points
		126–150: 5 points
		151–200: 7.5 points
		> 200: 9.5 points
Hemoglobin (mg/dL)	–	≥ 12.0: 0 points
		11.0–11.9: 1 point
		< 11.0: 1.5 points
WBC (× 10^3^/mm^3^)	–	≤ 12.0: 0 points
		> 12.0: 1.5 points
Albumin (mg/dL)	> 3.5: 0 points	> 3.8: 0 points
	≤ 3.5: 2 points	3.3–3.8: 1 point
		< 3.3: 1.5 points
Magnesium (mg/dL)	–	≥ 2.0: 0 points
		< 2.0: 1 point

Acute kidney injury (AKI) was defined as the degree of increase in serum creatinine levels from baseline

*RRT* Renal replacement therapy, *CDDP* Cisplatin, *WBC* White blood cell count

The ultimate goal of such clinical prediction models is to inform clinical decision-making in real-world practice. To enable this, external validation is essential to confirm that a model performs reliably in populations or settings different from those in which it was originally developed, whether geographically or temporally [13, 14]. However, to date, neither of these C-AKI prediction models has been evaluated outside Western populations [5, 15], including the Japanese population. The Motwani and Gupta models were developed using different outcome definitions. Therefore, directly comparing various outcome thresholds may offer valuable insights into their suitability for clinical applications. This study aimed to evaluate the performance of C-AKI prediction models developed in the US in Japanese populations and, based on the results, to identify issues in introducing these models into clinical practice in Japan, necessary adjustments, and future directions for model development.

## Methods

This study followed the Transparent Reporting of a Multivariable Prediction Model for Individual Prognosis or Diagnosis + artificial intelligence guidelines [16]. For full compliance details, please refer to the complete checklist presented in Table S1. The patients and members of the public were not involved in the design or conduct of this study. As this was a retrospective analysis of existing clinical data without any prospective intervention, no formal study protocol was prepared, and the study was not registered in a public trial registry. All statistical analyses were performed using the R version 4.3.1. OpenAI's GPT-4o language model was utilised to assist in the development and refinement of the R code used for these analyses. The code used for the data analysis is provided in Table S2. Statistical significance was defined as *p* < 0.05.

### Data source

This study used data from patients who received CDDP at Iwate Medical University Hospital between April 2014 and December 2023. The exclusion criteria were as follows: (i) age < 18 years at the time of administration, (ii) CDDP outside the study period or at another institution, (iii) treatment with daily or weekly CDDP regimens, and (iv) missing baseline renal function or outcome data. All eligible cases were included, and no a priori sample size calculations were performed.

Data on patient characteristics (age, sex, height, and weight), smoking history, concomitant medications, dates and doses of CDDP administration, and relevant laboratory values (creatinine, albumin, white blood cells, platelets, haemoglobin, and magnesium) were extracted from the electronic medical records. The estimated renal glomerular filtration rate was calculated using the Japanese Society of Nephrology [17]. Diabetes and hypertension were defined based on the use of the corresponding medications. The baseline laboratory values were defined as the most recent measurements obtained within 30 days of CDDP administration. Missing values were supplemented using regression-based imputation. Missing values were supplemented using regression-based imputation. Assuming missing at random, this method offers comparable predictive performance to multiple imputation in clinical prediction models and is a pragmatic alternative, especially as complete case analysis would drastically reduce the sample size, compromising generalisability [18]. However, regression-based imputation is not without limitations and may introduce bias, particularly with a high proportion of missing data.

### Definition of outcomes

In this study, C-AKI was defined as an increase in serum creatinine levels within 14 days of CDDP exposure. Based on the Kidney Disease: Improving Global Outcomes (KDIGO) criteria [19], C-AKI was defined as either an increase of ≥ 0.3 mg/dL or a ≥ 1.5-fold rise from baseline. Although an increase of ≥ 0.3 mg/dL over 14 days is a slight deviation from the strict KDIGO definition, this criterion was included to align with the outcome definition used in the Motwani model [12]. Severe C-AKI was defined as a ≥ 2.0-fold increase from baseline or the initiation of RRT (KDIGO stage ≥ 2).

### Calculation of scores and predictive probabilities

The scores were calculated according to the scoring criteria listed in Table 1. For the Motwani model, predictive probabilities were derived using the baseline risk and odds ratio reported in their development cohort—specifically, an incidence rate of 0.04 at a score of 0, and an odds ratio of 1.49 per one-point increase in score. As the simple model by Gupta et al. does not provide predictive probabilities for individual scores, the predictive probabilities were estimated using the primary model, which incorporates a cubic spline. The codes used in the calculations are listed in Table S2.

### Statistical performance

The statistical performance of the clinical prediction models was evaluated from three key perspectives: discrimination, calibration, and overall model fit [14].

Discriminatory ability, the capacity of the model to distinguish between individuals who do and do not experience an event, was assessed using the area under the receiver operating characteristic curve (AUROC). Differences between the two AUROCs were tested using the bootstrap method.

Calibration, which reflects the agreement between the predicted and observed risks, was evaluated using the following metric: calibration-in-the-large, which indicates systematic over- or underestimation across the model. The ideal value is 0; values < 0 suggest an overestimation, whereas values > 0 suggest an underestimation. The calibration slope represents the steepness of the calibration curve. A slope of 1 indicates perfect calibration, and values < 1 indicate extreme predictions in certain probability ranges.

The model fit was assessed using the Brier score, which quantifies the mean squared difference between the predicted probabilities and observed outcomes. A lower Brier score indicates a better model performance, with 0 being ideal.

While these performance metrics (the mean and 95% confidence intervals (CI) were estimated) were quantified using 1,000 bootstrap samples, the calibration plots (Figs. 3 and S1) were generated from the observed data for visual representation.

### Risk stratification with the gupta simple model

In the original study by Gupta et al., a simple model score was evaluated to stratify patients into risk groups, with accompanying expert recommendations on clinical management. By contrast, the Motwani et al. model does not propose a risk stratification system or clinical action guidelines. Therefore, we conducted an additional risk stratification analysis based on the Gupta model to explore how the risk grouping and associated clinical recommendations proposed in the original study were applied to our cohort. Patients were categorised into four groups according to their total score following the definitions from the original study as follows: low (0–5.5 points), moderate (6–9.5 points), high (10–15.5 points), and very high (≥ 16 points). The incidence of severe C-AKI was calculated for each group.

### Recalibration

Recalibration is a model-updating technique that adjusts regression coefficients to reflect the target population better [20]. Logistic recalibration was performed for each scoring system [21]. Specifically, the intercept and slope were re-estimated using logistic regression with the outcome as the dependent variable and each prediction score as the independent variable. Recalibration did not enhance the discriminative ability because the model structure remained unchanged. Although structural updates could potentially lead to substantial improvements in model performance, model updating was limited to recalibration given that this study was based on a single-centre cohort.

### Clinical utility

Decision curve analysis (DCA) was performed to evaluate the clinical utility of each model. In DCA, the net benefit, defined as the benefit of correct classification minus the harm of misclassification, is plotted on the vertical axis, whereas the threshold probability, the probability at which the benefits of treatment and non-treatment are considered equal, is shown on the horizontal axis. At any given threshold probability, a higher net benefit indicated greater clinical usefulness. Conversely, a negative net benefit suggests that model-guided decision-making may cause more harm than benefits.

## Results

**Fig. 1:**
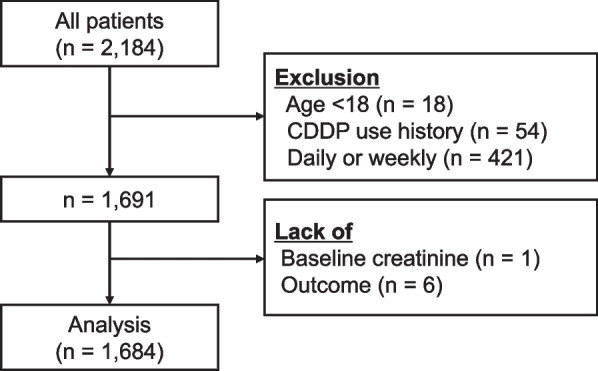
Flow diagram of patient selection. From an initial cohort of 2,184 patients who received cisplatin (CDDP), 493 were excluded based on age, prior CDDP use, or weekly or daily regimens. After further exclusion due to missing baseline creatinine or outcome data, 1,684 patients were included in the final analysis

A total of 2,184 patients who received CDDP during the study period were identified. After applying the exclusion criteria, 1,684 patients were included in the final analysis (Fig. 1). Patient characteristics and outcome frequencies are summarised in Table 2. The primary outcome, C-AKI, was observed in 186 (11.0%) patients, and severe C-AKI occurred in 36 (2.1%) patients.

**Table 2:** Patients’ background

	Saito et al (*n* = 1,684)	Gupta et al (*n* = 11,766)	Motwani et al (*n* = 2,128)
Study duration	2014–2023	2006–2022	2005–2014
Age, years	66 (58–71)	59 (50–67)	56.8 (13.2)
Male	1,124 (66.7)	6,935 (58.9)	1,173 (55.4)
BMI, kg/m^2^	21.9 (19.5–24.4)	–	26.6 (5.7)
CDDP dosage, mg	110 (90–130)	90 (60–160)	118.4 (77–151)
Hypertension	595 (35.3)	2,900 (24.6)	1,046 (49.4)
Diabetes	203 (12.1)	1,426 (12.1)	300 (14.2)
Smoker	1033 (64.8)	6,762 (57.5)	–
Creatinine, mg/dL	0.75 (0.63–0.87)	0.9 (0.7–1.0)	0.9 (0.2)
eGFR, mL/min/1.73 m^2^	74.0 (63.2–87.3)	90.0 (75.0–101.0)	87.3 (19.4)
WBC, × 10^3^/mm^3^	5.99 (4.64–7.56)	7.1 (5.6–9.1)	–
PLT, × 10^3^/mm^3^	256 (203–313)	255 (204–322)	–
Hemoglobin, mg/dL	12.5 (11.3–13.6)	12.8 (11.4–14.0)	–
Albumin, g/dL	3.9 (3.5–4.2)	4.1 (3.7–4.4)	4.0 (0.5)
Magnesium, mg/dL	2.05 (2.00–2.10)	2.19 (1.94–2.19)	–
C-AKI	186 (11.0)	–	289 (13.6)
Stage 1	150 (8.9)	–	234 (11.0)
Stage ≥ 2	36 (2.1)	608 (5.2)	55 (2.6)

Patient background characteristics and outcomes in our cohort as well as those reported in previous studies have been summarised. Continuous variables are presented as medians (interquartile ranges), and categorical variables are presented as n (%). In a study by Motwani et al., continuous variables, except for CDDP dose, are reported as means (standard deviations). The proportions of missing data in our cohort were as follows: albumin (6.0%); magnesium (61.2%); platelets (0.1%); and white blood cells (0.7%)

*BMI* Body mass index, *CDDP* Cisplatin, *eGFR* Estimated glomerular filtration rate, *WBC* White blood cell count, *PLT* Platelet count, *C-AKI* Cisplatin-associated acute kidney injury

**Fig. 2:**
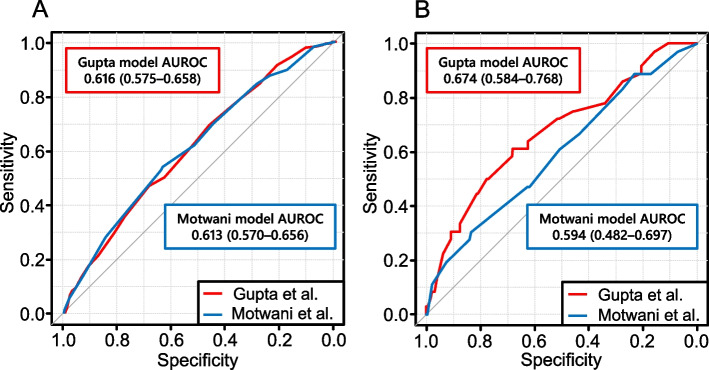
Receiver operating characteristic (ROC) curves for the prediction of cisplatin-associated acute kidney injury (C-AKI). The red and blue lines represent the ROC curves of the Gupta et al. and Motwani et al. models, respectively. Panels **A** and **B** represent patients with C-AKI and severe C-AKI, respectively

**Fig. 3:**
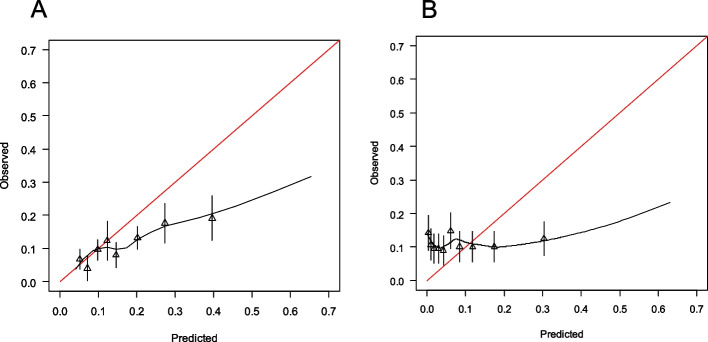
Calibration curves of the prediction models. The horizontal axis represents the predicted probability, and the vertical axis shows the observed incidence of cisplatin-associated acute kidney injury (C-AKI). Panels **A** and **B** correspond to the Motwani and Gupta models, respectively. The cohort was divided into ten risk-based groups, and the observed incidence for each group was plotted as triangles. The curves represent the smoothed regression lines. The red diagonal lines indicate perfect agreement between the predicted and observed probabilities. The calibration curves for severe C-AKI are shown in Fig. S2

The statistical performances of the clinical prediction models are summarised in Table 3, with a full comparison of all metrics provided in Table S3. Receiver operating characteristic (ROC) curves and calibration plots are presented in Figs. 2 and 3. The discriminatory performances of the Gupta and Motwani models for C-AKI showed no significant difference (AUROC, 0.616 [95% CI, 0.575–0.658] vs. 0.613 [95% CI, 0.570–0.656]; *p* = 0.84). By contrast, the Gupta model demonstrated significantly better discrimination of severe C-AKI (AUROC, 0.674 [95% CI, 0.584–0.768] vs. 0.594 [95% CI, 0.482–0.697]; *p* = 0.02). The calibration slopes and other indices revealed that both models were poorly calibrated.

**Table 3:** Indication parameter of model validation

	Motwani model	After recalibration	Gupta model	After recalibration
C-AKI				
AUROC	0.613 (0.570–0.656)	0.613 (0.570–0.656)	0.616 (0.575–0.658)	0.616 (0.575–0.658)
CITL	− 0.438 (–0.604– − 0.283)	− 0.004 (− 0.161–0.147)	0.346 (0.157–0.526)	− 0.003 (− 0.150–0.138)
Slope	0.550 (0.340–0.758)	1.006 (0.621–1.374)	− 0.075 (− 0.155–0.035)	0.995 (0.664–1.375)
Brier score	0.102 (0.092–0.112)	0.096 (0.085–0.108)	0.108 (0.095–0.119)	0.096 (0.086–0.107)
Severe C–AKI				
AUROC	0.594 (0.482–0.697)	0.594 (0.482–0.697)	0.674 (0.584–0.768)	0.674 (0.584–0.768)
CITL	− 2.242 (− 2.602– − 1.921)	− 0.023 (− 0.403–0.283)	− 1.567 (− 1.921– − 1.237)	− 0.018 (− 0.388–0.304)
Slope	0.523 (0.090–1.008)	0.993 (0.057–1.972)	− 0.005 (− 0.178–0.469)	0.996 (0.485–1.495)
Brier score	0.047 (0.042–0.052)	0.021 (0.014–0.027)	0.032 (0.027–0.038)	0.021 (0.014–0.027)

The mean values and 95% confidence intervals from the bootstrap samples are reported for each performance indicator. The parameters after recalibration are shown in the right-hand column, along with the corresponding original model indicators

*C-AKI* Cisplatin-associated acute kidney injury, *AUROC* Area under the receiver operating characteristic curve, *CITL* Calibration-in-the-large, *Slope* Calibration slope

**Fig. 4:**
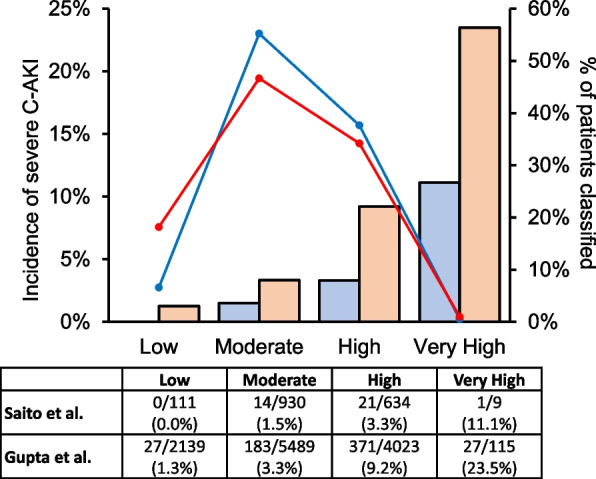
Comparison of risk stratification profiles between the original Gupta model and our cohort. Comparison of the distribution of patients and incidence of severe cisplatin-associated acute kidney injury (C-AKI) across risk groups defined by the Gupta simple model score. The blue bars and lines represent our cohort, whereas the red bars and lines represent the original developmental cohort. Patients were categorised into four risk groups (low [score 0–5.5], moderate [score 6–9.5], high [score 10–15.5], and very high [score ≥16]). The right axis shows the proportion of patients in each group, and the left axis shows the observed incidence of severe C-AKI. The numbers in the table indicate the number of patients with severe C-AKI compared to the total number of patients in each risk group

In addition, risk stratification based on the Gupta simple model score is shown in Fig. 4. This risk stratification analysis was exploratory and intended to illustrate the distribution of risk categories in our cohort rather than serving as a primary evaluation of model performance. Although the model demonstrated reasonably consistent separation between risk groups, the overall risk distribution was lower than that of the original development cohort, with most patients categorised into moderate- or high-risk groups.

**Fig. 5:**
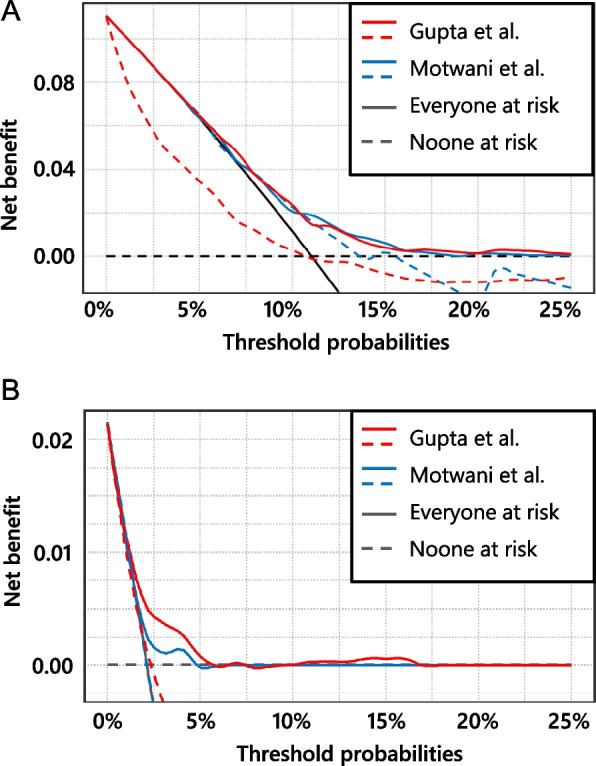
Decision curve analysis of the prediction models. The net benefits for the prediction models developed by Motwani et al. and Gupta et al. are plotted across a range of threshold probabilities. Panel A displays results for patients with cisplatin-associated acute kidney injury (C-AKI), and Panel **B** displays results for patients with severe C-AKI. In both panels, dashed lines represent the models before recalibration, while solid lines represent the recalibrated versions. The threshold probability represents the point at which the benefits of interventions to prevent C-AKI outweigh the risks of those interventions. In simpler terms, a lower threshold probability (towards the left of the graph) indicates a greater emphasis on preventing nephrotoxicity compared to the potential benefits of treatment. Once a clinically relevant threshold probability is established, a superior model will demonstrate higher net benefits (i.e., a higher position on the graph)

Logistic recalibration was performed to adjust both models to the characteristics of our cohort; the updated coefficients are presented in Table S4. The clinical utility was evaluated using DCA for both the original and recalibrated models (Fig. 5). For C-AKI, the non-recalibrated Gupta model (dashed red line) demonstrated lower net benefit than the model-independent strategy (black line) across most threshold probabilities, whereas the non-recalibrated Motwani model (dashed blue line) yielded net harm at higher thresholds (approximately ≥ 12.5%). By contrast, both recalibrated models exhibited positive net benefits across a wide range of thresholds with comparable clinical utility. A similar pattern was observed for severe C-AKI; both uncalibrated models showed net harm, which improved after recalibration. The recalibrated Gupta model demonstrated the highest net benefit for predicting severe C-AKI.

## Discussion

In this study, we conducted the first external validation of two existing clinical prediction models for C-AKI developed by Motwani et al. and Gupta et al. in a Japanese patient population. The discriminative ability of the two models for C-AKI was comparable. However, the Gupta model demonstrated superior performance in predicting severe C-AKI, which was clinically significant. Both models initially exhibited poor calibration, and the DCA further indicated that using unadjusted models for clinical decision making did not produce a net benefit. After logistic recalibration, both models showed improved clinical utility, with the recalibrated Gupta model offering the greatest net benefit to patients with severe C-AKI. These results highlight the importance of recalibrating the prediction models before their application to new populations, particularly in different geographic and clinical contexts.

Clinical prediction models are typically optimised using development cohorts and perform poorly when applied to different populations. Discriminatory performance declined when the Motwani and Gupta models were applied to the Japanese cohort. White individuals accounted for approximately 80% of participants in the original development cohorts [5, 12], suggesting that these models were primarily trained on non-Asian populations. Demographic differences, along with variations in healthcare delivery systems and baseline risk profiles, may have contributed to the reduced discrimination. Additionally, compared to the original studies, our cohort was older, had a smaller body size, and exhibited less variability in CDDP dosing (Table 2). This relative homogeneity in patient characteristics and risk profiles may have constrained the ability of the models to distinguish between patients who did and did not develop C-AKI [22].

Another potential source of performance variation is the differences in the definitions of AKI between studies. The Motwani model used C-AKI, whereas the Gupta model targeted severe C-AKI as the primary outcome (Table 1). None of the models was originally evaluated for transportability across different outcome definitions. Predicting severe AKI is particularly relevant for guiding treatment decisions because it is associated with worse renal prognosis and overall survival [12, 23, 24]. However, even modest elevations in creatinine levels may affect renal outcomes [25]. Therefore, differences in outcome definitions must be carefully considered when interpreting the model performance across populations.

The decline in calibration performance was more pronounced than the reduction in discriminatory ability. Calibration, which reflects the agreement between the predicted and observed risks, is critical for the reliable clinical application of prediction models. Recent studies have consistently reported that models developed in Western populations show greater discrepancies in calibration than discrimination when applied to Asian settings [26–28]. Similar to discrimination issues, this may be attributed to demographic differences, healthcare system variations, baseline patient characteristics, and differences in the definitions of C-AKI. Moreover, differences in serum creatinine measurement methods between US populations (often the Jaffe method) and Japan (enzymatic method, which generally yields lower values) could also contribute to discrepancies in baseline creatinine values and subsequent AKI definitions, thereby impacting calibration performance.

Poor calibration can be particularly detrimental when clinical decisions are based on predicted risk estimates [29]. Underestimation may lead to missed cases of C-AKI, whereas overestimation may prompt unnecessary therapeutic interventions, such as switching from cisplatin to alternatives (e.g. carboplatin or oxaliplatin [30]), reducing chemotherapy doses, or intensifying supportive care. These interventions can increase healthcare costs and compromise treatment efficacy. The DCA (Fig. 5) showed that both models, prior to recalibration, provided either no net benefit or net harm across a wide range of clinically relevant threshold probabilities. Furthermore, the negative CITL observed in both models before recalibration suggests that the models overestimate the risk of C-AKI (Table 3). Altogether, these findings suggest that directly applying the original models without adjustment may lead to inappropriate decision-making in Japanese clinical practice.

In the present study, we attempted model recalibration to improve the performance. Although recalibration often improves calibration, it is not expected to enhance discrimination. In our analysis, recalibration improved both the calibration and the clinical utility of the Motwani and Gupta models. This improvement may be attributed to the fact that both models retained a certain level of discriminatory ability despite being applied to different populations. However, the cutoffs for continuous variables and the weighting of individual predictors in existing models were originally optimised for development cohorts using data-driven approaches. Consequently, these thresholds and score ratios may not be optimal for Japanese clinical settings. Furthermore, an AUROC < 0.7 may be considered insufficient performance, and this insufficient discrimination suggests the presence of important unmeasured predictors. As our study was based on a single-centre cohort, model updating was limited to recalibration. Future efforts should aim for more comprehensive model updates, such as incorporating additional predictors, refining cutoff values, and validating across multiple centres, to enhance the applicability and accuracy of prediction models in Japanese clinical practice.

Despite these needs for future refinement, the recalibrated models, particularly the Gupta model for severe C-AKI, offer valuable insights for informing clinical decisions in the present. Gupta et al. present expert opinions on clinical management for each stratified risk category [5]. Specifically, for moderate risk (approximately 5% probability of severe C-AKI), they recommend avoiding nephrotoxic drugs (such as nonsteroidal anti-inflammatory drugs or contrast agents). For high or very-high risk (10% or higher probability of severe C-AKI), they recommend considering using alternative platinum agents, reducing CDDP dosage, increasing monitoring frequency, and increasing hydration volume. However, as discussed earlier, discrepancies in predicted probabilities may lead to excessive interventions. Therefore, clinical decisions should be based on the absolute probability expected in that cohort. In our cohort, the probability of severe C-AKI was approximately 5% for high risk and approximately 10% for very high risk. Clinical interventions corresponding to these predicted values will be considered. Furthermore, improved local calibration is crucial for facilitating shared decision-making. By providing patients with more accurate, population-specific risk probabilities, clinicians can foster informed discussions, personalize care plans, and align interventions with patient values, thereby enhancing active participation in their care.

This study has some limitations. First, the representativeness of the study population is limited. Owing to data availability, external validation was conducted using a single-centre cohort from a university hospital located in a rural area, where the prevalence of hypertension is higher than that in urban settings [31]. In addition, our cohort may be skewed towards older patients because of regional demographic characteristics. These factors could have influenced both baseline risk and model performance, underscoring the need for cautious generalisation of our findings. Second, the proportion of missing magnesium values (61.2%) was high. Although regression-based imputation was used, we acknowledge that such a high degree of missingness (61.2% for magnesium) can inherently reduce the accuracy of imputed values and may introduce potential bias, especially for a key predictor in the Gupta model, potentially leading to an underestimation of the model’s true performance. Indeed, a sensitivity analysis revealed that excluding serum magnesium significantly lowered the model's AUROC (*p* = 0.003, Fig. S2), underscoring its substantial contribution. As magnesium measurement is not technically difficult, routine assessment of serum magnesium levels is recommended when applying the Gupta model in future clinical settings. Third, no a priori sample size calculations were conducted. Although the commonly cited criterion of 100 events for model validation has been met [32], the recent literature suggests more precise sample size recommendations for external validation studies [33]. Based on these updated criteria (the parameters and results in this calculation are shown in Table S5), the sample size in this study may have been insufficient to ensure precise calibration estimates. Therefore, conclusions regarding the calibration should be interpreted with caution. However, the consistent observation of poor initial calibration for both models strongly suggests a qualitative lack of agreement between predicted and observed risks, irrespective of the imprecision in exact estimates. Lastly, the stability of recalibration estimates must be taken into consideration, as logistic recalibration involves estimating regression coefficients. While recalibration inherently limits the risk of overfitting compared to structural model updates, we acknowledge that the recalibration parameters, particularly for severe C-AKI, might be less stable due to the small number of events (36 events/2 parameters). Although this potential for reduced precision should be considered when generalizing these specific recalibrated parameters, the event-per-parameter ratio of 18 for severe C-AKI is considered borderline but acceptable, meeting minimum criteria. Future research should aim to validate and refine these prediction models using larger and more diverse cohorts, ideally through national databases or multicentre observational studies, to enhance their generalisability and clinical utility in Japanese practice.

## Conclusions

To the best of our knowledge, this study is the first to externally validate the Motwani and Gupta models for predicting C-AKI in a Japanese population. Although both models demonstrated modest discriminatory performance, the Gupta model showed superior ability to identify patients at risk for severe C-AKI, a clinically important outcome. However, both models demonstrated poor initial calibration, limiting their direct clinical utility. Importantly, recalibration substantially improved both calibration and clinical utility, underscoring the need for population-specific model adaptation prior to clinical application. These findings highlight the potential utility of existing models in the Japanese setting, but only after appropriate recalibration. Although the models showed modest discriminatory performance, their recalibrated forms may support risk-informed decision-making when interpreted alongside clinical judgment. Future research should focus on model refinement through structural updates and validation in large multicentre Japanese cohorts to support reliable, individualised risk stratification and optimise the safe use of cisplatin in clinical practice.

## Supplementary Information

Supplementary Material 1.

## Data Availability

Data supporting the findings of this study are available upon request from the corresponding author. The data are not publicly available because of privacy and ethical restrictions.

## Abbreviations

AKI: Acute kidney injury
AUROC: Area under the receiver operating characteristic curve
BMI: Body mass index
C-AKI: Cisplatin-associated acute kidney injury
CDDP: Cisplatin
CI: Confidence interval
CITL: Calibration-in-the-large
DCA: Decision curve analysis
eGFR: Estimated glomerular filtration rate
KDIGO: Kidney Disease: Improving Global Outcomes
PLT: Platelet count
ROC: Receiver operating characteristic
RRT: Renal replacement therapy
WBC: White blood cell count
