# Supplementary material for: External validation and comparison of clinical prediction models for cisplatin-associated acute kidney injury: a single-centre retrospective study
Source: J Pharm Health Care Sci. 2025 Sep 29;11:82. doi: 10.1186/s40780-025-00471-0 (PMC12482746; doi:10.1186/s40780-025-00471-0)
Supplement: Supplementary file 1 — Supplementary Material 1. [file 40780_2025_471_MOESM1_ESM.docx]

**Supplymental material**

**External validation of clinical prediction rules for cisplatin-associated acute kidney injury: A single-centre retrospective study evaluating multiple treatment cycles**

Kazuki Saito, Satoru Nihei, Junichi Asaka, Kenzo Kudo

**Table S1.** TRIPOD + AI checklist

**Table S2.** R code used in this study

**Table S3.** All indication parameters of model validation

**Table S4.** Recalibrated regression coefficients

**Table S5.** Sample size calculation

**Fig. S1.** Calibration curves for severe and mild C-AKI

**Fig S2.** Comparison of AUROC between the original Gupta model and the model excluding serum magnesium

**Table S1**. TRIPOD+AI checklist

| Item No. | Checklist Item | Corresponding Section / Paragraph |
| --- | --- | --- |
| Title |  |  |
| 1 | Identify the study as developing or evaluating the performance of a multivariable prediction model, the target population, and the outcome to be predicted | Title |
| Abstract |  |  |
| 2 | See TRIPOD+AI for Abstracts checklist | N/A (Abstract not provided) |
| Introduction |  |  |
| 3a | Explain the healthcare context (including whether diagnostic or prognostic) and rationale for developing or evaluating the prediction model, including references to existing models | Introduction par.1 |
| 3b | Describe the target population and the intended purpose of the prediction model in the context of the care pathway, including its intended users (e.g., healthcare professionals, patients, public) | Introduction par.2 |
| 3c | Describe any known health inequalities between sociodemographic groups | N/A |
| 4 | Specify the study objectives, including whether the study describes the development or validation of a prediction model (or both) | Introduction par.3 |
| Methods |  |  |
| 5a | Describe the sources of data separately for the development and evaluation datasets (e.g., randomised trial, cohort, routine care or registry data), the rationale for using these data, and representativeness of the data | Materials and Methods > Data source par.1 |
| 5b | Specify the dates of the collected participant data, including start and end of participant accrual; and, if applicable, end of follow-up | Materials and Methods > Data source par.1 |
| 6a | Specify key elements of the study setting (e.g., primary care, secondary care, general population) including the number and location of centres | Materials and Methods > Data source par.1 |
| 6b | Describe the eligibility criteria for study participants | Materials and Methods > Data source par.1 |
| 6c | Give details of any treatments received, and how they were handled during model development or evaluation, if relevant | Materials and Methods > Data source par.1 |
| 7 | Describe any data pre-processing and quality checking, including whether this was similar across relevant sociodemographic groups | Materials and Methods > Data source par.2 |
| 8a | Clearly define the outcome that is being predicted and the time horizon, including how and when assessed, the rationale for choosing this outcome, and whether the method of outcome assessment is consistent across sociodemographic groups | Materials and Methods > Definition of Outcomes par.1 |
| 8b | If outcome assessment requires subjective interpretation, describe the qualifications and demographic characteristics of the outcome assessors | N/A |
| 8c | Report any actions to blind assessment of the outcome to be predicted | N/A |
| 9b | Clearly define all predictors, including how and when they were measured (and any actions to blind assessment of predictors for the outcome and other predictors) | Materials and Methods > Data source par.2 |
| 9c | If predictor measurement requires subjective interpretation, describe the qualifications and demographic characteristics of the predictor assessors | N/A |
| 10 | Explain how the study size was arrived at (separately for development and evaluation), and justify that the study size was sufficient to answer the research question. Include details of any sample size calculation | Discussion par.10 |
| 11 | Describe how missing data were handled. Provide reasons for omitting any data | Materials and Methods > Data source par.2 |
| 12d | Describe if and how any heterogeneity in estimates of model parameter values and model performance was handled and quantified across clusters (e.g., hospitals, countries). See TRIPOD-Cluster for additional considerations | N/A (single-centre study) |
| 12e | Specify all measures and plots used (and their rationale) to evaluate model performance (e.g., discrimination, calibration, clinical utility) and, if relevant, to compare multiple models | Materials and Methods > Statistical Performance par.2 |
| 12f | Describe any model updating (e.g., recalibration) arising from the model evaluation, either overall or for particular sociodemographic groups or settings | Materials and Methods > Recalibration par.1 |
| 12g | For model evaluation, describe how the model predictions were calculated (e.g., formula, code, object, application programming interface) | Materials and Methods > Calculation of Scores par.2, Table S2 |
| 13 | If class imbalance methods were used, state why and how this was done, and any subsequent methods to recalibrate the model or the model predictions | N/A (Not explicitly addressed) |
| 14 | Describe any approaches that were used to address model fairness and their rationale | Discussion par.2 (limited mention of generalizability) |
| 16 | Identify any differences between the development and evaluation data in healthcare setting, eligibility criteria, outcome, and predictors | Discussion par.2 |
| 17 | Name the institutional research board or ethics committee that approved the study and describe the participant-informed consent or the ethics committee waiver of informed consent | Materials and Methods > Ethical statement par.1 |
| Open science | |  |
| 18a | Give the source of funding and the role of the funders for the present study | Funding |
| 18b | Declare any conflicts of interest and financial disclosures for all authors | Conflicts of interest |
| 18c | Indicate where the study protocol can be accessed or state that a protocol was not prepared | Materials and Methods > par.1 |
| 18d | Provide registration information for the study, including register name and registration number, or state that the study was not registered | Materials and Methods > par.1 |
| 18e | Provide details of the availability of the study data | Data availability |
| 18f | Provide details of the availability of the analytical code | Materials and Methods > Calculation of Scores par.2, Table S2 |
| Patient and public involvement | |  |
| 19 | Provide details of any patient and public involvement during the design, conduct, reporting, interpretation, or dissemination of the study or state no involvement | Materials and Methods > par.1 |
| Results |  |  |
| 20a | Describe the flow of participants through the study, including the number of participants with and without the outcome and, if applicable, a summary of the follow-up time. A diagram may be helpful | Results par.1, Fig. 1 |
| 20b | Report the characteristics overall and, where applicable, for each data source or setting, including the key dates, key predictors (including demographics), treatments received, sample size, number of outcome events, follow-up time, and amount of missing data. A table may be helpful. Report any differences across key demographic groups | Results par.1, Table 2 |
| 20c | For model evaluation, show a comparison with the development data of the distribution of important predictors (demographics, predictors, and outcome). | Table 2 |
| 21 | Specify the number of participants and outcome events in each analysis (e.g., for model development, hyperparameter tuning, model evaluation) | Results par.1, Table 2 |
| 23a | Report model performance estimates with confidence intervals, including for any key subgroups (e.g., sociodemographic). Consider plots to aid presentation | Results par.2, Table 3, Fig. 2 and 3 |
| 23b | If examined, report results of any heterogeneity in model performance across clusters. See TRIPOD Cluster for additional details | N/A (single-center) |
| 24 | Report the results from any model updating, including the updated model and subsequent performance | Results par.3, Table S4 |
| Discussion |  |  |
| 25 | Give an overall interpretation of the main results, including issues of fairness in the context of the objectives and previous studies | Discussion par.1 |
| 26 | Discuss any limitations of the study (such as a non-representative sample, sample size, overfitting, missing data) and their effects on any biases, statistical uncertainty, and generalizability | Discussion par.5 |
| 27c | Discuss any next steps for future research, with a specific view to applicability and generalizability of the model | Discussion par.6 |

**Table S2.** R code used in this study

| R package | **#Calliblation plot**; library(CalibrationCurves); val.prob.ci.2  **#AUROC**; library(pROC); auc.ci & roc.test  **#Desicion curve analysis**; library(dcurves); dca |
| --- | --- |
| Customed | **#Calicirate liner predictor (Gupta et al. primary model)**  calculate_primary_model <- function(df) {  df <- df %>%  mutate(  primary_model = -3.686659 +  0.04421425 * Age +  0.0000183485 * pmax(0, Age - 30)^3 - 0.0002163227 * pmax(0, Age - 51)^3 +  0.0004394909 * pmax(0, Age - 59)^3 - 0.0002907307 * pmax(0, Age - 66)^3 +  0.0000492141 * pmax(0, Age - 76)^3 -  (0.2525115 * Mg -  5.801969 * pmax(0, Mg - 1.7)^3 + 29.89521 * pmax(0, Mg - 1.9456)^3 -  43.75054 * pmax(0, Mg - 2.1)^3 + 18.23788 * pmax(0, Mg - 2.2)^3 +  1.419414 * pmax(0, Mg - 2.432)^3) -  (0.04471118 * Hb -  0.009984642 * pmax(0, Hb - 9.1)^3 + 0.07499566 * pmax(0, Hb - 11.6)^3 -  0.1355953 * pmax(0, Hb - 12.8)^3 + 0.08595369 * pmax(0, Hb - 13.9)^3 -  0.01536942 * pmax(0, Hb - 15.5)^3) +  0.01993736 * WBC -  0.003237126 * pmax(0, WBC - 3.8)^3 + 0.02176998 * pmax(0, WBC - 5.8)^3 -  0.03178413 * pmax(0, WBC - 7.1)^3 + 0.01411246 * pmax(0, WBC - 8.8)^3 -  0.0008611787 * pmax(0, WBC - 14.5)^3 -  (0.4990617 * ALB -  0.02681548 * pmax(0, ALB - 3)^3 + 4.312953 * pmax(0, ALB - 3.8)^3 -  13.50495 * pmax(0, ALB - 4.1)^3 + 10.66724 * pmax(0, ALB - 4.3)^3 -  1.448432 * pmax(0, ALB - 4.7)^3) +  0.04708185 * Dose -  0.00001028397 * pmax(0, Dose - 38)^3 + 0.00002391644 * pmax(0, Dose - 65)^3 -  0.00001521614 * pmax(0, Dose - 90)^3 + 0.000002039042 * pmax(0, Dose - 150)^3 -  0.0000004553695 * pmax(0, Dose - 220)^3 +  0.00397566 * PLT -  0.0000005145004 * pmax(0, PLT - 137)^3 + 0.00000222745 * pmax(0, PLT - 210)^3 -  0.000002459005 * pmax(0, PLT - 255)^3 + 0.0000007631055 * pmax(0, PLT - 312)^3 -  0.00000001705063 * pmax(0, PLT - 488)^3 -  (4.626802 * Scr +  94.00696 * pmax(0, Scr - 0.6)^3 - 187.9561 * pmax(0, Scr - 0.7)^3 +  232.7567 * pmax(0, Scr - 0.9)^3 - 153.7796 * pmax(0, Scr - 1.0)^3 +  14.97206 * pmax(0, Scr - 1.3)^3) +  0.2739952 * HT + 0.2528551 * DM  )  return(df)  }  **#Callibration parameters (bootstrapped)**  bootstrap_metrics <- function(data, indices) {  d <- data[indices]  logit_model <- tryCatch(lrm(outcome ~ predicted, data = d), error = function(e) return(NA_real_))  if (inherits(logit_model, "try-error")) {  cox_snell_r2 <- NA_real_  nagelkerke_r2 <- NA_real_  } else {  cox_snell_r2 <- as.numeric(logit_model$stats["R2"])  nagelkerke_r2 <- as.numeric(logit_model$stats["Dxy"]) / 2  }  brier_score <- mean((d$predicted - d$outcome)^2)  calib_large <- tryCatch(  coef(glm(outcome ~ offset(log(predicted/(1 - predicted))), family = binomial, data = d))[1],  error = function(e) NA_real_  )  obs_exp_ratio <- mean(d$outcome) / mean(d$predicted)  calib_model <- glm(AKI20 ~ I(log(predicted / (1 - predicted))), family = binomial, data = d)  calib_slope <- coef(calib_model)[2]  ici <- mean(abs(d$outcome - d$predicted))  return(c(nagelkerke_r2, cox_snell_r2, brier_score, calib_large, obs_exp_ratio, calib_slope, ici))  }  B <- 1000  boot_results <- boot(data, bootstrap_metrics, R = B) |

**Table S3**. All indication parameters of model validation

|  | Motwani et al. | After recalibration | Gupta et al. | After recalibration |
| --- | --- | --- | --- | --- |
| C-AKI |  |  |  |  |
| AUROC | 0.613 (0.570-0.656) | 0.613 (0.570-0.656) | 0.616 (0.575-0.658) | 0.616 (0.575-0.658) |
| Nagelkerke R² | 0.112 (0.068-0.153) | 0.113 (0.069-0.154) | 0.013 (-0.025-0.055) | 0.115 (0.073-0.158) |
| Cox-Snell R² | 0.030 (0.001-0.057) | 0.032 (0.012-0.057) | 0.002 (0.000-0.008) | 0.034 (0.014-0.062) |
| Brier Score | 0.102 (0.092-0.112) | 0.096 (0.085-0.108) | 0.108 (0.095-0.119) | 0.096 (0.086-0.107) |
| Calibration-in-the-large | -0.438 (-0.604--0.283) | -0.004 (-0.161-0.147) | 0.346 (0.157-0.526) | -0.003 (-0.150-0.138) |
| Observed / Expected | 0.702 (0.610-0.797) | 0.999 (0.868-1.136) | 1.318 (1.134-1.513) | 0.999 (0.877-1.127) |
| Calibration Slope | 0.550 (0.340-0.758) | 1.006 (0.621-1.374) | -0.075 (-0.155-0.035) | 0.995 (0.664-1.375) |
| Integrated Calibration Index (ICI) | 0.225 (0.215-0.235) | 0.193 (0.181-0.205) | 0.176 (0.163-0.189) | 0.193 (0.182-0.204) |
| Severe C-AKI |  |  |  |  |
| AUROC | 0.594 (0.482-0.697) | 0.594 (0.482-0.697) | 0.674 (0.584-0.768) | 0.674 (0.584-0.768) |
| Nagelkerke R² | 0.096 (0.009-0.189) | 0.095 (0.003-0.193) | 0.066 (-0.022-0.171) | 0.172 (0.072-0.268) |
| Cox-Snell R² | 0.022 (0.001-0.067) | 0.022 (0.000-0.067) | 0.019 (0.000-0.063) | 0.046 (0.007-0.102) |
| Brier Score | 0.047 (0.042-0.052) | 0.021 (0.014-0.027) | 0.032 (0.027-0.038) | 0.021 (0.014-0.027) |
| Calibration-in-the-large | -2.242 (-2.602--1.921) | -0.023 (-0.403-0.283) | -1.567 (-1.921--1.237) | -0.018 (-0.388-0.304) |
| Observed / Expected | 0.136 (0.095-0.183) | 0.992 (0.674-1.316) | 0.252 (0.179-0.341) | 0.097 (0.686-1.340) |
| Calibration Slope | 0.523 (0.090-1.008) | 0.993 (0.057-1.972) | -0.005 (-0.178-0.469) | 0.996 (0.485-1.495) |
| Integrated Calibration Index (ICI) | 0.170 (0.164-0.177) | 0.042 (0.035-0.048) | 0.100 (0.094-0.108) | 0.041 (0.035-0.048) |

**Table S4.** Recalibrated regression coefficients

|  | Motwani et al. | | Gupta et al. | |
| --- | --- | --- | --- | --- |
|  | β_0_ | β_1_ | β_0_ | β_1_ |
| C-AKI | -2.8787 | 0.2216 | -3.8451 | 0.1835 |
| Severe C-AKI | -4.5752 | 0.2073 | -6.5772 | 0.2767 |

liner predictor = β_0_ + β_1_×Score (per one point)

**Table S5.** Sample size calculation

| **Parameter** | Motwani | Gupta |
| --- | --- | --- |
| Event ratio | 10% | 10% |
| C-statistic | 0.7 | 0.7 |
| O/E ratio CI width | 0.22 | 0.22 |
| Srope CI width | 0.3 | 0.3 |
| C-statistics CI width | 0.1 | 0.1 |
| Liner predictor, mean (SD) | -1.86 (0.74) | -3.14 (1.75) |
| **Calculated sample size** | |  |
| O/E ratio | 2860 | 2860 |
| Slope | 2945 | 1264 |
| C-statistics | 1154 | 1154 |

**Fig. S1.** Calibration curves for severe C-AKI

The left panels display results for Motwani et al. model, and the right panels for Gupta et al. model.


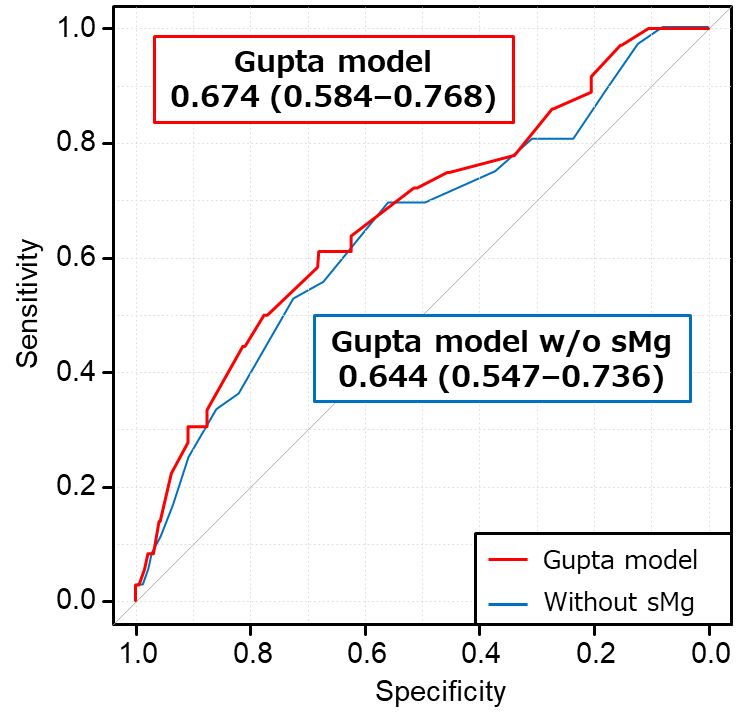


**Fig S2.** Comparison of AUROC between the original Gupta model and the model excluding serum magnesium

The figure compares the AUROC of the original Gupta model with that of a model that did not include serum magnesium (w/o sMg) to determine the extent of sMg's contribution to severe C-AKI prediction. The AUROC was significantly lower in the model that did not include sMg (p=0.003).
